# HEALTH AND AGING POLICY FELLOWSHIP: AN INCREDIBLE JOURNEY

**DOI:** 10.1093/geroni/igae098.0201

**Published:** 2024-12-31

**Authors:** Natalie Baker

**Affiliations:** University of Alabama at Birmingham School of Nursing, Birmingham, Alabama, United States

## Abstract

Dr. Natalie Baker is a geriatric nurse practitioner and nursing professor. Her passion for improving the quality of care delivered in the long-term care setting led her to apply for the Health and Aging Policy Fellowship (HAPF). She will describe her previous national experience in health policy and desire to gain an in-depth immersion experience in policymaking at the federal level. As a nonresidential HAPF with the U.S. Senate Special Committee on Aging, Dr. Baker has had the opportunity to interact with national aging policymakers, research topics of concern, assist in drafting legislative bills, and plan committee hearings. Through the HAPF program, she experienced an unforgettable opportunity to have conversations with affected individuals, health care workers, and professional organizations. She observed skillful negotiations for bipartisan support in a deeply divided Congress while working alongside Senator Bob Casey’s staff. From this incredible journey, Dr. Baker has gained a new perspective on aging challenges and pathways for meeting the needs of this vulnerable population.

